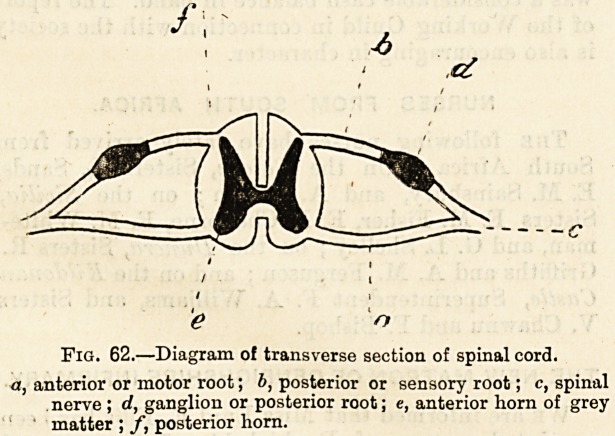# The Hospital. Nursing Section

**Published:** 1902-10-18

**Authors:** 


### The Hospital.

### Nursing Section.

Contributions for this Section of "The Hospital" should be addressed to the Editor, "The Hospital" Nursing Section, 28 & 29 Southampton Street, Strand, London, W.C.

No. 838.—Vol. XXXIII. *SATURDAY, OCTOBER* 18, 1902.

### Notes on News from the Nursing World.

### QUEEN ALEXANDRA'S IMPERIAL MILITARY NURSING SERVICE.

In another part of this paper will be found the Regulations for admission to Queen Alexandra's Imperial Military Nursing Service, which have been sent to us by the matron-in-chief. We give them without any abbreviation in order that each of our readers may possess a copy for future reference, and also because of the interest which they will undoubtedly excite. It is just a year ago since the committee appointed by the Secretary for War to consider the reorganisation of the Army and Indian Nursing Service, issued their scheme. The Regulations now promulgated show that there have been several more or less important departures from the recommendations embodied in the report. For example, the principal matron, instead of starting with a salary of £110 a year, rising to £160, is to receive £150 at the outset which will be increased to £180. The staff nurses, instead of beginning with a salary of £25, as recommended by the report, will receive £30. With regard to the admission of the staff nurse, the recommendation was that she should be on probation for three months, but in the Regulations the period is six months. The allowance for annual clothing in a home station is £8 instead of £6, and abroad £9 instead of £7. The duties of both matrons and sisters are considerably amplified. Rule 161, we are glad to see, makes the sister responsible for "the cleanliness, ventilation, lighting, warming, as well as good order, of her wards and annexes." The Regulations are evidently the result of the most careful consideration by experts, and the introduction of staff nurses has entailed some entirely new rules. One effect of the changes set forth in the Regulations will be to bring the Military Nursing Service more into line with the general hospitals.

### THE SCOTTISH QUEEN VICTORIA MEMORIAL FUND.

The appeal which has been issued by the Duchess of Buccleuch as president of the Scottish branch of Queen Victoria's Jubilee Institute, is not intended to interfere with the Women's Memorial Fund. It represents an organised effort which is being made throughout each Scottish county with the view of raising the sum of £30,000, to be be used for work in Scotland only. There is at present a deficit of £600 a year, and this has to be met annually out of a small amount of capital at the disposal of the Scottish Council. It is in order to put things on a sound financial basis that the appeal of the Duchess has been addressed to "the men, women, and children in Scotland." Very wisely, too, her Grace is starting a "Shilling Fund" in every county, and is inviting the co-operation of schools, institutions, golf, football, and cricket clubs in this laudable undertaking. We venture to predict that, if the "Shilling Fund" is worked with energy and system, it will be a very important auxiliary.

### OUR CHRISTMAS DISTRIBUTION.

An early response has been made to our appeal to our readers to send us as soon as possible useful articles of clothing for the patients in hospitals and infirmaries, for distribution in the Christmas season. We acknowledge the receipt of parcels from Miss A. Cecil, Farm Avenue, Streatham, and Madame Laczkovic, No. 37, R.N.P.F.N., with all the more pleasure, because we hope that the promptness of the senders will impel others to favour us with their contributions quickly. In view of the exhibition of the articles which we propose to hold before they are despatched to their destination, it is particularly important that our friends should lose no time in forwarding their parcels. In all cases they should be addressed to the Editor, 28 & 29 Southampton Street, Strand, London, and should be marked "Clothing Distribution."

### SUPPLY AND DEMAND IN SOUTH AFRICA.

The warning which we gave last week that, although trained nurses in South Africa are well paid, the demand might not keep pace with the increase in the supply, has been speedily justified. Miss Borlase, Honorary Secretary and Matron of the Nurses' Co operative Society, Johannesburg, writing to us under date of September 19th, says that the Co operative Society's list is closed, and that she is getting letters at the rate of 30 per mail, to which it is impossible for her to reply individually. Miss Borlase adds that when the mines are in full work, and the country is developed, there may be an increased demand, but at present there are no openings. The matron of the Victoria Nurses' Institute, Hof Street, Capetown, writes to us in a similar strain, and states that there are many nurses in Capetown now with absolutely nothing to do. She also is so inundated with letters from England that she asks us to intimate that it is useless under existing conditions to apply to her for employment, though, "no doubt later on, when the country is more settled, there will be work for more nurses." The plethora of nurses does not, we believe, so far apply to Kimberley and some of the coast towns.

### RESIGNATION OF A LONDON MATRON.

The post of matron of the North-Eastern Hospital for Children will, we understand, be vacant at the end of the present year, and applications from ladies willing to undertake the duties will shortly be invited. The present matron, Miss E. W. Curno, who retires after twenty-five years' service, has done most valuable work, and has always commanded the respect and affection both of her nurses and of all associated with her. Her departure from the institution in Hackney Road will be deeply and generally regretted.

### AN AGE QUESTION IN PARIS.

As the result of a petition by the parents, the French Government have resolved for the present to leave the care of the infirmaries in State colleges and lycées to the nuns. The plea in favour of this course urged by the parents was twofold. It was alleged that the lay nurses are incompletely trained, and that "youths at an impressionable age are more suitably cared for by elderly nurses belonging to families of the aristocracy or higher bourgeoisie." The proper answer to the first allegation would obviously be to employ completely trained lay nurses, but as yet the supply is, we conclude, insufficient. With regard to the second, it suggests that on the other side of the Channel the practice of fixing a hard-and-fast age-limit does not find favour. There are unquestionably on both sides patients of the male sex who may be nursed with advantage by women no longer in their first youth.

### CONFERENCE OF DISTRICT NURSING SUPERINTENDENTS.

From a rather belated report which has been sent to us, of the first annual conference of the Metropolitan and South of England District Nursing Superintendent held at the Midwives Institute, we learn that among the subjects discussed were, How to enlist the sympathy of matrons of training schools in order to recruit nurses; Daily visiting and paying cases; the necessity of exciting local interest and how best to do it; the night nursing difficulty, and the question of district nurses' salaries. As to the first it was decided to ask for the help and co-operation of all hospital matrons and to invite the attendance of four annually at each conference. On the question of payment by patients, the conclusion arrived at was that each committee must decide for itself according to local needs; while among the means of exciting local interest, house-to-house collections, entertainments on a large scale, and thanksgiving services for patients and nurses, found favour. Among the proposals for solving the night nursing difficulty, it was suggested that one member of each staff should be told off for night duty and provided with a bicycle. As to salaries, the majority of members seemed, we are informed, to think that as the opportunities for rising in district posts are very limited, the rate of remuneration should be somewhat higher than that of hospital nurses.

### THE IRISH LOCAL GOVERNMENT BOARD AND WORKHOUSE NURSING.

In a report just issued by the Irish Local Government Board, reviewing the question of nursing in Irish Workhouse Infirmaries and Hospitals, there are thefollowing most significant passages :—

It would appear to us to be worthy of most serious consideration whether this very important question of the nursing of the sick might not properly be taken up and dealt with in the United Kingdom by conference between the various teaching and administrative bodies concerned, so that substantial uniformity as regards instruction and qualifications might as far as possible be arranged for, and care be taken that hospitals, having no pretensions for giving adequate instruction to nurses, should not be permitted to issue certificates of training and competence that by too many are accepted as if such certificates were of equal value to those issued by the leading hospitals of the kingdom. If the heads of any comprehensive general scheme could be arrived at in conference, such a settlement could be confirmed or varied in Parliament.

A very large proportion of the present Poor Law nursing staff possess no qualifications whatever, except such experience and practice as they acquired since their appointment, and their only instruction has been that given by the Visiting Medical Officer on the occasion of his daily visit or visits—in some cases extended and careful, but in others of a perfunctory character as regards giving systematic instruction to nurses. Since the passing of the Local Government (Ireland) Act, 1898, it has been our practice to decline to sanction the appointment of any untrained or uncertified person as a nurse or assistant nurse; and our opinion is that no person should be appointed, even temporarily, to discharge nursing duties until it has been certified that she or he possesses the qualifications of a medical and surgical nurse.

### STATE REGISTRATION IN AMERICA.

A meeting will be held in Rochester, New York State, on the 31st of this month, for the purpose of discussing the prospects of legislation as a means of raising the standard of nursing. The members of the New York State Nurses' Association are of opinion that the best way of preventing people who have not received professional training from serving as trained nurses, is to obtain State registration for trained nurses. But there is another side of the question which will hardly be lost sight of by the American Legislature. To some extent State registration might hinder unqualified persons from passing themselves off as qualified, but it would never do to accept the State certificate as the one and only passport to confidence. What guarantee could the State give that a nurse who had been registered for 10 years was still capable of discharging her duties ? Yet, however unfit in character she had proved herself for the work, she would be able, by means of the proof that she was registered, to produce a credential sufficient to trap the unwary. Recent references will always be essential to a nurse's success.

### "GRATUITIES" AT ST. GERMANS.

A short time ago the St. Germans Board of Guardians decided at their usual meeting to give their two late nurses—Miss Ivey and Miss Owen—£30 and £20 respectively as gratuities. Naturally, the announcement was received with much satisfaction by the nurses concerned. But this was considerably qualified when, on receipt of their cheques, they found that the "gratuities" included their quarter's salary, and were also given conditionally on the understanding that they made no claim on account of the amount they had paid last year towards their superannuation allowance. In these circumstances, it is clear that the Guardians abused the use of the word "gratuity," and raised hopes which they did not intend to fulfil.

### A QUEEN'S NURSE FOR TOTTENHAM.

The Chairman of the Edmonton Board of Guardians proposed at the last meeting that the Board should contribute £10 towards the cost of providing a Queen's nurse for the Tewkesbury district of Tottenham. He said that it was a very poor district where the ministration of a Queen's nurse would be a great boon. The proposal found so much favour that another member of the Board suggested that £20 should be granted. It was, however, considered more judicious to start with the smaller amount, and we do not think that the Guardians can be accused of niggardliness in deciding to limit their contribution to £10. Edmonton covers a large acreage, with an increasing number of poor in almost every district; and if, as it seems likely, each district desires to possess a Queen's nurse, it will expect to be treated on the same footing as Tewkesbury.

### HOSPITAL ECONOMICS.

An interesting announcement is made by the authorities of the Columbia University in New York. At the Teachers' College a course in hospital economics is given, which has for its purpose the preparation of trained nurses who have the necessary qualification for teachers in training schools for nurses. The aim is eventually to attain uniformity in curriculum and training school methods, which shall make the standing of a trained nurse practically the same for every training school connected with a general hospital in the United States, and also in the course of time to be able to supply thoroughly trained superintendents to take charge of hospitals and training schools. The importance of nurses having a thorough knowledge of hospital economics is indisputable, and we hope, on a future occasion, to supply some interesting details of the course of instruction which is offered at the New York College in the science of household management.

### CITY ORTHOPÆDIC HOSPITAL.

The annual course of free lectures to the nurses at the City Orthopædic Hospital will be commenced on Friday evening at 4.30 p.m., and continued fortnightly, with a short break at Christmas, until May 13th, 1903. Mr. Noble Smith will commence the series, his subject being "The Nursing of Orthopædic Cases," and on the 31st inst. he will deal with "The Management of Curvatures of the Spine." Mr. John Poland gives ten lectures on "The Human Skeleton"; Mr. Chisholm Williams takes "The Heart and Circulation" first, and then "The Stomach and Digestion"; while Mr. Jackson Clarke has selected "The Preparation of Patients for Operation," and "The Nurse's Duties in the Operating Theatre." Mr. Noble Smith's third and fourth lectures will be on "The Care of Patients after Operation," and "Common Errors in Surgical Nursing"; Mr. John Poland will also lecture on "The Muscles" and "Regional Anatomy"; Mr. Chisholm Williams on "Bacteria, etc.," and "Antiseptics, Disinfectants"; and Mr. Jackson Clarke on "Bandaging" and "Splints." Altogether the series is both varied and useful.

### PROGRESS AT PECKHAM.

The second annual meeting of the Peckham Nursing Association was by no means conventional in character. It took place in the Peckham Public Hall, was presided over by the Mayor of Camberwell, the company included the local clergy, doctors, and other well known persons. The hall was tastefully decorated with fairy lamps, flowers, and plants. Before the business of the evening was transacted refreshments were served, and after they were concluded there was a concert. The report shows a material increase in the work, and gave point to the statement that the services of another nurse will shortly be required. A sum of £60 has already been set aside to form a nucleus of a fund for the establishment of a nurses' home, and the tone of the speeches encourages the belief that there will be a liberal response to the urgent appeal for a considerable increase in the number of subscribers.

### NURSING AT LARNE.

The financial position of the charities in connection with the Larne District Nursing Society is satisfactory. The society itself commenced the year with a balance of £11 and finished it with a surplus of £39. The number of visits, 2,507, paid during the year was considerably less than the previous year. This does not appear to have been accounted for by any failure on the part of the nurses, as it was stated that in every case where illness had been reported to the nurse, or that the assistance of the society had been sought, the case had been attended to, and that, if it was considered a suitable one, a certain amount of necessary nourishment had also been allowed. The society contributed in the year towards the keep of a patient in the Samaritan Hospital, who had had to undergo an operation. The report of the workings of "The Annie Clark Trust for Incurables" showed that the nurse had paid 983 visits since the last report, and that there was a considerable cash balance in hand. The report of the Working Guild in connection with the society is also encouraging in character.

### NURSES FROM SOUTH AFRICA.

The following nurses have lately arrived from South Africa:—On the *Galeka*, Sisters L. Sands, E. M. Sainsbury, and A. Sutton; on the *Sicilia*, Sisters E. M. Fisher, E. L. Chaborne, E. M. Whiteman, and G. L. Shelley; on the *Dunera*, Sisters R. Griffiths and A. M. Ferguson; and on the *Kildonan Castle*, Superintendent F. A. Williams, and Sisters V. Chawnu and F. Bishop.

### THE NEW MATRON OF DENBIGHSHIRE INFIRMARY.

We are informed that Miss Enid E. Ellis has been appointed matron of Denbighshire Infirmary, and that she was for five years head nurse in the same institution. The experience of Miss Ellis, it is stated, ranges over a term of ten years, and during the last nine months she has been attached to the Elgin Nursing Institution, Maida Vale, London.

### SHORT ITEMS.

Lady Jerningham, whose death occurred last week, was one of the vice-presidents of the Berwick and District Nursing Association, and took a great interest in its work.—In commemoration of the Coronation the nursing staff of the Granville Road Institution, Newcastle-on-Tyne, have presented the matron with a gold watch and the assistant matron with a gold brooch set with sapphires and diamonds.

### Lectures to Nurses on Anatomy.

By W. Johnson Smith, F.R.C.S., Principal Medical Officer, Seamen's Hospital, Greenwich.

### LECTURE XXVIII.—THE ORGANS OF THE NERVOUS SYSTEM.—(*Continued from page* 5.)

If either of the hemispheres be cut across just above the level of the connecting band called the *corpus callosum* the following important parts of the brain will be exposed:—(*a*) along the circumference a thin wavy border of grey matter—the cortex of the brain—covering the free surfaces of the convolutions and continued into the numerous fissures or *sulci* between the convolutions; within this (*b*) a broad oval surface of white matter presenting a strong contrast to the dark matter on the surface; (*c*) near the inner part of this white surface an irregularly shaped cavity —the *lateral ventricle*, extending a little outwards in front to form what is termed the *anterior cornu*, and continued behind into a much longer extremity hooked inwards which is termed the *posterior cornu*; (*d*) on the floor of the lateral ventricles two large elevations composed mostly of grey matter; that in front known as the *corpus striatum*, the larger one behind and to the inner side as the *optic thalamus*. The first of these elevations or cerebral ganglia takes an important part in the transmission to the body of motor impulses, and the latter, the *optic thalamus*, in the transmission to the brain of sensory impulses.

The lateral ventricles in the right and left hemispheres open at a deeper level into a single cavity called the *third ventricle*, which again communicates by a narrow canal called the *fissure of Sylvius* with another single cavity—the *fourth ventricle*—situated at the back of the bulb. These ventricles, which contain a thin and limpid fluid — the cerebro-spinal fluid—are dilatations of a single central canal extending from the brain to the lower part of the spinal

Fig. 62.—Diagram of transverse section of spinal cord.

*a*, anterior or motor root; *b*, posterior or sensory root; *c*, spinal nerve; *d*, ganglion or posterior root; *e*, anterior horn of grey matter; *f*, posterior horn.

cord. Deeply placed in the brain between the anterior horns of the lateral ventricles is another small cavity, or rather interspace, completely shut in, which is called the *fifth ventricle*.

Between the posterior parts of the two lateral ventricles above and the upper part of the cord below there is a very complex arrangement of ganglionic masses of grey matter and of connecting nerve fibres, constituting what is known as the mid and hind brains. The most important of the ganglions are situated in the fourth ventricle behind the bulb, from which region, as has been already stated, most of the cranial nerves are derived.

*The spinal cord*, which is continuous with the medulla oblongata, does not occupy the whole length of the spinal canal. In the adult it terminates at the level of the first lumbar vertebra, the inferior portion of the canal being occupied by a large mass of nerves, which is called the *cauda equina* or horse's tail. It is not, like the brain, closely packed within its bony investment, but hangs loosely in the canal, being anchored to its walls by special ligamentous structures, and by the nerves which pass from it on either side through the intervertebral foramena to the different parts of the body. Like the brain it has its three membranes, *dura-mater*, *pia-mater*, and *arachnoid*, the first forming a loose sac or sheath. The cord varies in thickness at different parts, and presents two distinct enlargements, one at the lower part of the neck, the other in the back, which correspond respectively to the nerves given off at these portions to the upper and lower limbs.

The diagram (Fig. 62) of a transverse section of the spinal chord is intended to show the following points:—

1. The relations of white and grey matter differ in the cord and brain. In the latter, we have seen, the grey matter is on the surface; in the cord, on the other hand, the white matter is on the outside, and the grey matter in the interior, where it forms a double crescent, the inner or convex curves of which are joined in the centre of the cord. The anterior extremities of these two curves or crescents are thicker and shorter than the posterior limbs. The two portions of each crescent beyond their junction in the centre of the cord are called the *cornua* or horns, that in front being the *anterior* and that behind the *posterior horn*. The tip of the longer and narrower posterior horn approaches closely to the surface of the cord and is there continuous on either side with nerve-fibres. Between the end of the anterior horn and the front of the cord there is a perceptible margin of white mattter.

2. On each side of the cord there are two thick bundles of nerve-fibres—the *anterior and the posterior roots*. These two coalesce and form a single nerve (spinal nerve), which leaves the spinal canal by the corresponding intervertebral foramen, and serves either alone or in association with spinal nerves given off above and below it to supply a definite portion of the body. Of the spinal nerves given off along the whole length of the cord there are 31 pairs. We should note one important point of difference between the root behind and that in front. The former, it will be seen, is thickened before it joins the former and presents a distinct and regular swelling which is termed the *ganglion of the posterior root*.

3. The cord, like the cerebrum, is incompletely divided into two lateral halves, but presents two fissures instead of a single one; the anterior fissure being wider than, though not so deep as, the posterior one.

Before we bring to an ending our brief review of the organs of the nervous system, we ought to obtain some idea of the paths followed by the motor and the sensory nerves in their uninterrupted course between the brain and the parts of the body—mainly muscles and skin—to which they are distributed. The physiological fact already mentioned to you, that each hemisphere of the brain governs exclusively, not the same, but the opposite side of the body, is explained by a crossing of the motor nerve fibres from one side to the other near the anterior surface of the bulb. Some sensory fibres also cross, or *decussate*, some at the lower part of the bulb and others in the cord at different levels. The anterior roots of the spinal cord, which have just been described, are made up exclusively of nerves of motion, and each posterior root, which, as we have seen, presents a distinct swelling or ganglion, contains only sensory or afferent fibres. The spinal nerve formed by the junction of these roots after it

### LECTURES TO NURSES ON ANATOMY.—*Continued.*

has passed beyond the spine through its corresponding intervertebral foramen divides into an anterior or ventral branch which subdivides into branches supplied to the front of the body, and a posterior or dorsal branch for the posterior part. The anterior branches supplied to the limbs form by their subdivisions and interlacements complex networks called plexuses, that including the nerves of the upper limb being called the *brachial plexus*, and the two by which the lower limb receives its different nerves the *lumbar* and *sacral* plexuses.

The *brachial plexus* which is formed by interlacing of the anterior branches of the fifth, sixth, seventh, and eighth cervical, and the first thoracic nerve, is concentrated in the armpit into the following long and distinct nerves which supply the arm, forearm, and hand:—

(*a*) The *external cutaneous*, both motor and sensory, which is distributed to the flexor muscles above the elbow and the skin on the outer part of the forearm.

(*b*) The *median* (also motor and sensory) distributed to most of the flexor muscles below the elbow and the skin of the palm of the hand and the front of the thumb and some of the fingers.

(*c*) The *ulnar* supplies two flexor muscles in the forearm and the skin in the outer part of the hand both back and front, and the little and half the ring fingers.

(*d*) The *musculo-spiral*, the largest branch of the plexus, supplies the extensor muscles above the elbow, and the skin on the back of the arm and of the outer part of the back of the hand.

(*e*) The *internal cutaneous*, which is a purely sensory nerve, supplies the skin of the inner half of the upper limb below the elbow.

### The Stormy Side of Burgher Camp Life.

BY A DAY-SISTER IN SOUTH AFRICA.

A long time ago we were warned to expect rain in September, but all the same the storm of the 11th came as an unwelcome shock. It had been a heavy oppressive day, with a hot wind and plenty of dust blowing. Suddenly, about 5 P.M., the wind ceased in a very ominous fashion, the sky grew wonderfully dark, then came a huge dust-storm, followed by thunder, incessant lightning, and hail. Without exaggeration the stones were as large as hens' eggs, and if they came in contact with an uncovered head an attack of concussion seemed imminent. Our marquees were cheerfully pitched on a mealie-field with the result that in a very short time the floors were inches deep in churned-up brown water, while the newly constructed sloots, converted themselves into spruits. Alas! those were not our only troubles, for the tent-pegs went flying gaily, and before 8 P.M. only two marquees and a few bell-tents were left standing.

### Housing the Homeless.

Our doctors and superintendents worked splendidly, assisted by the matrons and most of the sisters. Luckily, a permanent building had been erected for pneumonia cases, and although the beds were full we had to accommodate nearly all the patients from the marquees. The children were packed in "herring" fashion, and the bigger ones reposed on mattresses on the floor, while a few convalescents were fixed up in the kitchen. Carrying sick folk through a slough of despond, with a storm raging furiously is no easy task, but before nine o'clock we had them all settled in dry garments, and, under the circumstances, fairly comfortable.

### Half-drowned Nurses.

Then we began to think a little about ourselves. The outlook was far from cheerful, for we were literally without a bed among us. However, the relief matrons had a cottage in the camp, and they threw open their doors in the most hospitable fashion to the weary, homeless, half-drowned hospital folk, providing us with beds, dry garments, hot tea, and all sorts of luxuries. Our hospital matron and the doctors heroically refused to leave, and spent a more than dreary night in the hospital kitchen with exemplary fortitude. When we day-sisters returned to the hospital ground in the early morning, the working gang were struggling to dig the marquees out of the mud, and the state of our beds and garments can be better imagined than described. A stiff wind was still blowing, and some of my chattels floated gaily on the breeze into the spruit half a mile away. Most of us rather resembled a collection of rag-pickers in mackintosh coats and storm-boots than civilised Englishwomen. Our mess-table had been laid for dinner when the marquee collapsed, and when the canvas was raised great was the ruin therein. After diligent search various treasures were excavated, and among others a black-handled "three-pronger" and several knives which hardly cut hot butter. The Boers took everything very stolidly, while the Kaffir boys regarded it all as a huge joke. Saturday was a wet drizzly day, and gave us no chance to dry our chattels, but Sunday's sun shone cheerfully on a drying ground, mattresses, rugs, hats, etc., all spread out as at a jumble sale.

### A Disastrous Thunderstorm.

Fortunately none of the patients appeared any the worse, and as our sufferings have been confined to the loss of tooth-brushes, garments, pictures, etc., we have much to be thankfu for. Three Boers returning from the station on the night of the storm were struck by lightning, two being killed instantaneously, while the other, a woman, was extensively burnt and is at present a patient in our hospital. The camp orphanage was one of the first places to collapse, and late at night one of our doctors discovered two small youths peacefully slumbering beneath the ruins. The camp people are accommodated almost entirely in bell-tents and these stood their ground uncommonly well, very few collapsing. Our hospital camp is beginning to present a fairly respectable appearance, and we now tramp over hard ruts instead of bogs, but none of us will ever forget September 11th, 1902.

### To Nurses.

We invite contributions from any of our readers, and shall be glad to pay for "Notes on News from the Nursing World," or for articles describing nursing experiences, or dealing with any nursing question from an original point of view. The minimum payment for contributions is 5s., but we welcome interesting contributions of a column, or a page, in length. It may be added that notices of appointments, entertainments, presentations, and deaths are not paid for, but that we are always glad to receive them. All rejected manuscripts are returned in due course, and all payments for manuscripts used are made as early as possible after the beginning of each quarter.

### Queen Alexandra's Imperial Military Nursing Service.

### REGULATIONS FOR ADMISSION.

### I.—CONSTITUTION.

1. The Queen Alexandra's Imperial Military Nursing Service shall consist of :—(1) A matron-in-chief ; (2) principal matrons ; (3) matrons ; (4) sisteis (5) staff nurses ; (6) such non-commissioned officers and first-class orderlies of the Royal Army Medical Corps as have been specially recommended.

### II.—APPOINTMENT AND QUALIFICATION OF CANDIDATES.

2. Appointments to the Queen Alexandra's Imperial Military Nursing Service shall be given to persons duly qualified, in accordance with the following regulations :—

3. A candidate for the position of staff nurse or sister must be between 25 and 35 years of age, single or a widow, and possess a certificate of not less than three years' training and service in medical and surgical nursing in a civil hospital recognised by the Advisory Board. She must be of British parentage or a naturalised British subject. The matron-in-chief will be required to satisfy the Nursing Board that, as regards education, character, and social status, she is a fit person to be admitted to Queen Alexandra's Imperial Military Nursing Service. The candidate will be required to fill in a declaration which will be forwarded to her on application, and to produce the following documents :—

(*a*) A certificate of registration of birth ; or, if this is not obtainable, a declaration made before a magistrate by one of her parents or former guardians, giving the date of her birth.

(*b*.) Recommendations from the matrons of the civil hospital at which she was trained, and of that at which she is serving at time of application.

4. Before being appointed, the candidate will appear before a sub-committee of the Nursing Board, which will make recommendations as to her appointment. Should her appointment be decided upon, arrangements will be made for her physical examination.

### III.—TERMS OF APPOINTMENT.

5. *Staff Nurses.*—If accepted for service, a staff nurse will be engaged for a period of two years, during the first six months of which her appointment is of a provisional character. A special report will be made at the end of the provisional period, and another on the conclusion of her engagement, as to the staff nurse's work, conduct, and suitability in all respects for the Queen Alexandra's Imperial Military Nursing Service. These special reports will be made by the matron of the hospital, and will be forwarded to the matron-in-chief through the officer in charge of the hospital, to be laid before the Nursing Board.

6. *Sisters and Matrons.*—Sisters and matrons will be recommended by the Nursing Board on the advice of the matron-in-chief.

### IV.—DRESS.

7. Members of the Queen Alexandra's Imperial Military Nursing Service will provide themselves with the following uniform :—

Matron-in-Chief.

Grey uniform, faced with scarlet, and braided. Scarlet cape.

Principal Matrons and Matrons.*

*Annual*—1 grey serge dress, 2 grey alpaca dresses (with scarlet cuffs), 6 muslin caps, 6 turned-down collars, 6 pairs turned-back cuffs, 2 scarlet capes, and 1 grey bonnet.

*Triennial*—1 summer cloak, grey, with scarlet collar, and 1 winter cloak, grey, with scarlet collar.

Sisters.

*Annual*—1 grey serge dress, 3 grey washing dresses (with two scarlet bands, 1 inch wide, on the cuffs), 6 muslin caps, 6 turned-down collars, 6 pairs turned-back cuffs, 2 scarlet capes, 1 grey bonnet, and 8 aprons.

*Triennial*—1 summer cloak and 1 winter cloak.

Staff Nurses.

*Annual*—1 grey serge dress, 3 grey washing dresses, 6 muslin caps, 6 collars, 6 pairs turned-back cuffs, 2 scarlet capes, 1 grey bonnet, and 8 aprons.

*Triennial*—1 summer cloak and 1 winter cloak.

Helmets, or white sailor hats, with plain distinctive ribbon bands, may be worn when serving in hot climates or in the country. Detailed particulars will be furnished by the matron-in-chief on application.

In uniform, ornaments are not to be worn.

8. The uniform will be purchased by the members of the Service themselves, an allowance for this purpose being granted under paragraph 690, Allowance Regulations (*see* p. 6).

The establishment selected to supply it will be intimated to them on application to the matron-in-chief.

The accounts and vouchers from each firm of uniform supplied will be kept by the matrons for inspection by the matron-in-chief.

### V.—PAY, PENSIONS, AND ALLOWANCES.

(*Extracts from the Pay Warrant.*)

Pay.*

"682b. The pay of our Queen Alexandra's Imperial Military Nursing Service shall be as follows :—

| —— | Initial Rate. | | | Annual Increment. | | | Maximum. |
|---|---|---|---|---|---|---|---|
| | £ | s. | d. | £ | s. | d. | £ |
| Matron-in-chief... | 250 | 0 | 0 | 10 | 0 | 0 | 300 |
| Principal matron | 150 | 0 | 0 | 5 | 0 | 0 | 180 |
| Matron ... ... | 70 | 0 | 0 | 5 | 0 | 0 | 120 |
| Sister ... ... | 37 | 10 | 0 | 2 | 10 | 0 | 50 |
| Nurse ... ... | 30 | 0 | 0 | 2 | 10 | 0 | 35 |

"A matron may be granted charge-pay at a rate not exceeding £30 a year, according to the magnitude of her charge.

"682d. Pay may be issued in advance for a period not exceeding one month, prior to embarkation for service abroad.

"682e. The pay of the female servant appointed to attend on the nursing sisters at Netley and Woolwich shall be £25 a year, and at other hospitals £15 a year, rising by annual increments of £1 to £20 a year.

* In hospitals where matrons are required to nurse they will provide themselves with one grey alpaca dress instead of two, two grey washing dresses, and six aprons.

* In addition to the pay and allowances specified in this section, matrons, sisters, and nurses are supplied with public quarters, (or with lodgings at the public expense), and with fuel and light.

### QUEEN ALEXANDRA'S IMPERIAL MILITARY NURSING SERVICE—*Continued.*

Pay during Leave of Absence.

"682F. Pay during ordinary leave of absence may be granted in each financial year for the following periods:—

"Matron-in-chief, 6 weeks; principal matron, 6 weeks; matron, 6 weeks; sister, 5 weeks; nurse, 4 weeks.

"682G. Pay may be granted for accumulated leave of absence during service at a station abroad.

"682H. Pay during leave of absence on account of injury or sickness may be granted as under:—

"(*a.*) When the injury or sickness is certified by the regulated medical authority to have been caused by the Service, full pay may be issued for a period of 12 months, and half-pay for such further period as sick leave may be granted.

"(*b*) When the injury or sickness is not caused by the Service, full pay may be granted for a period of three months; and, after 20 years' service, two-thirds pay; or with less than 20 years' service, half-pay for a further period of three months. In special circumstances, and subject to the approval of the General Officer Commanding, pay at the reduced rate may be granted for a third period of three months.

"(*c*) When the sickness occurs at the station, a period not exceeding 30 days shall, if duly certified by the regulated medical authority, be excluded from the period of absence on ordinary leave to which the issue of pay is limited.

Retirement, Pensions, and Gratuities.

"682I. *Service as a nursing sister at a military hospital in the employ and pay of the National Aid Society, followed continuously by established service as a sister or nurse in Our Army Medical Service, may be allowed to count towards pension.*

"682J. A member of Our Queen Alexandra's Imperial Military Nursing Service may retire voluntarily on pension on attaining the age of 50, and shall be compulsorily retired at the age of 55.

"682K. If pensioned on account of disability, one year of service in a tropical climate may count as two years towards pension.

"682L. She shall be entitled to retire on pension after 10 years' service if she is rendered unfit for hospital duty through disease or injury, certified by the regulated medical authority to have been caused by the service.

"682M. She may at any time be required to retire on account of unfitness for the duties of her appointment, with such gratuity as she may be entitled to under Article 682R.

"682N. The pension shall be calculated on the rate of pay at the time of retirement, and shall, after 10 years' service, be 30 per cent. of such pay, with an additional 2 per cent. for each year of service in excess of 10, up to a maximum of 70 per cent. of such pay.

"In any case of special devotion to duty, a higher pension, not exceeding £50 a year, may be granted.

"682P. If disabled in the Service, after five but under 10 years' service, such rate of pension below that fixed in Article 682N shall be granted as may be determined by our Secretary of State. If she has served for less than five years when disabled, she shall receive a gratuity, to be determined in like manner.

"682R. A member of Our Queen Alexandra's Imperial Military Nursing Service retired under Article 682M may, provided she has not been guilty of misconduct, be granted a gratuity of one month's pay for each year of service, if not entitled to a pension under Article 682N.

"682S. In cases where a member of Our Queen Alexandra's Imperial Military Nursing Service is pensioned for a disability not permanently unfitting her for duty, the pension shall cease on the date when she again becomes fit for duty, unless there should then be no vacancy, in which case, should she be willing to continue her service, she may remain on pension for a period not exceeding one year, pending a vacancy.

"682T. A member of Our Queen Alexandra's Imperial Military Nursing Service retiring without having previously obtained permission to do so shall forfeit all claim to pension or gratuity."

(*Extract from the Allowance Regulations.*)

Allowances.

"690. An allowance in lieu of board and washing at the rate of 15s. a week at a home station, or of 21s. a week at a station abroad, will be granted to each member of the Queen Alexandra's Imperial Military Nursing Service. A special allowance for the provision of clothing will also be granted, except to the matron-in-chief, at the following rates:—Annual clothing and cloak allowance abroad, £9; annual clothing and cloak allowance at home, £8.

"An allowance of 10s. 6d. a week for board, etc., will be granted to the servant appointed to attend on the members of the Queen Alexandra's Imperial Military Nursing Service. The other allowances at stations abroad, including the allowance for servants, will be at such rates, not exceeding those of a departmental officer of subaltern rank, as the Secretary of State may determine."

### VI. DISCIPLINE AND DUTIES.

*Extracts from Regulations for Army Medical Services.*

Matron-in-Chief.

141. The Matron-in-Chief will be responsible for keeping the Service records and confidential reports from the matrons of the various hospitals regarding the character, conduct, and efficiency of the sisters and staff nurses under their control.

142. She will, by frequent inspections, keep herself acquainted with the administration of the Nursing Service in the various military hospitals.

143. She will submit to the Nursing Board recommendations for the appointment, promotion, distribution, retirement, and dismissal of members of the Service.

144. She will be responsible for maintaining a sufficient staff of special staff nurses, detailing them for duty in cases of emergency, or for service in smaller hospitals.

145. She will present every year to the Nursing Board a scheme for the annual leave of matrons and special staff nurses, and will report to the Board the arrangements made by matrons for the annual leave of sisters and staff nurses.

146. She will perform such other duties as may be from time to time determined by the Nursing Board.

Matrons.

147. Matrons will be responsible for the general nursing arrangements of the hospital, for the due performance of their duties by the sisters and staff nurses, and for the maintenance of good conduct, efficiency, and discipline amongst all members of the female nursing and domestic staff, as well as for the cleanliness and good order of the wards under their charge. She will not be responsible for nursing in wards which are set apart by the officer in charge of the hospital for cases which he may consider unsuitable for female nursing.

148. She will supervise the training of the non-commissioned officers and men of the nursing section of the Royal Army Medical Corps in nursing duties.

### QUEEN ALEXANDRA'S IMPERIAL MILITARY NURSING SERVICE—*Continued.*

149. She will be responsible to the officer in charge for demanding sufficient supply, for the good condition and cleanliness, of the bedding and linen in the nurses' quarters and the wards under her nursing charge.

149A. She will frequently inspect the equipment and bedding to ascertain whether any damage has been done thereto, and will check them with the inventories periodically.

150. She will see that proper medical and nursing attendance is provided without delay for sick members of the nursing or female domestic staff.

151. She will arrange the annual leave of sisters, staff nurses, and female domestic staff, reporting thereon through the officer in charge to the matron-in-chief.

152. In all instances of difficulty she will apply to the officer in charge, who will render her every assistance in the performance of her responsible duties.

153. She will see that all orders and instructions of the medical officers treating the cases are duly carried out by the sisters and staff nurses.

154. When she is informed of any neglect of duty or impropriety of conduct, whether on the part of sisters, staff nurses, non-commissioned officers or men of the Royal Army Medical Corps, patients, or visitors, she will at once report it to the officer in charge (*see* paragraph 161).

155. She will detail a sister or staff nurse to prepare instruments and dressings at operations, and to assist at all operations in sisters' wards.

156. She will take such share in nursing as ordered by the matron-in-chief.

157. She will keep the books and accounts connected with the nursing staff; and a monthly record of the messing will be kept, together with a statement of the cost, vouched by bills of expenditure; the special allowances drawn by the nursing staff under paragraph 690, allowance regulations, being entered in liquidation thereof. The register is intended as a permanent record, and will be vouched by the signature of the matron, and inspected periodically by the matron-in-chief at home, and by the principal medical officer in charge abroad.

158. She will fix the hours of duty, meals, and recreation for sisters and staff nurses, subject to instructions from the matron-in-chief, in such a manner as will comply with Garrison Standing Orders.

159. When a staff nurse or sister is transferred from one hospital to another, the matron will prepare a confidential report for transmission through the officer in charge for the information of the matron under whom the sister or staff nurse will serve. On, or as soon as possible after, January 1st a similar confidential report will be forwarded on all sisters and nurses through the officer in charge to the matron-in-chief.

### Sisters.

160. Every sister in a military hospital will be under the immediate supervision of the matron, and directly responsible to her in all matters relating to conduct and discipline. She will receive and carry out such orders and instructions relative to the treatment of the sick as she may receive from the officer in charge, whom she will accompany in his visits.

161. She will be responsible for the personal cleanliness of the patients in her wards, and for the cleanliness, ventilation, lighting, warming, as well as good order of her wards and annexes. Any neglect of duty, or impropriety of conduct, whether on the part of staff nurses, non-commissioned officers or orderlies, patients, or visitors, will be reported by her to the matron. In cases of emergency she will apply for the assistance of the orderly medical officer or non-commissioned officer on duty.

When in doubt or difficulty in any matter she will at once inform the matron, who will, if necessary, bring it to the notice of the officer in charge, or, in his absence, to the medical officer on duty.

161A. A sister is not permitted to accept presents of any kind from any patient, or friend of any patient, whether during his illness or after his death, recovery, or departure.

161B. A sister is not, at any time, to go to wards in which she is not working, except on special business; she is not to remain in her own wards, or visit in any other wards, when off duty.

A sister may not allow staff nurses or orderlies to visit in her wards, except on business, or by special leave of the matron.

161C. All talking in the wards, corridors, and on the stairs is strictly forbidden; a sister is required to be quiet and orderly when moving about the hospital.

161D. She is to adhere punctually to her time-tables, and to be most particular in returning to her wards at the exact time specified.

161E. Sisters are not to visit each other after 10.30 P.M., but must retire to their rooms by that hour, unless special permission for late leave be obtained. Their bedrooms are to be neat and orderly, and all lights are to be extinguished therein by 11 P.M., unless special permission be given.

161F. A sister is not to absent herself from meals without permission. Except at the recognised "off-duty" times she will not absent herself from the hospital or quarters without permission.

161G. Uniform is to be worn on all occasions, both indoors and out of doors, except when on leave out of garrison, or by special permission of the matron.

161H. Before going off duty each sister is required to put in writing on the night memorandum-sheet any notes on special cases, or other important matters which may be necessary for the guidance of the night staff nurses and orderlies, or which it may be desirable to bring to the notice of the night sister. The night sister shall see that these instructions are carefully carried out. She will record the hours of her visits to each ward, and will note on the night memorandum-sheet any information she may wish to bring to the notice of the ward sister. Similarly, the night sisters and staff nurses will record matters of importance for the information of the day sister.

162. In cases of fresh admissions into her ward she will ascertain when the patients last had any food, and see that they are not kept waiting for suitable nourishment. She must impress upon orderlies the importance of this duty.

162A. Sisters and orderlies should unite in showing special sympathy and kindness to the friends of those patients who are on the "dangerous list."

162B. When a death takes place the sister in charge of the ward will see that the body is reverently prepared for the mortuary, and will then inform the senior non-commissioned officer, who will proceed in accordance with para. 204, Standing Orders, Royal Army Medical Corps.

162C. When a patient is to be discharged, she will send him, together with his diet sheet and temperature chart, to the office of the Senior Medical Officer at the hour appointed.

162D. She will draw from the steward the personal equipment required for each patient on admission, and will be responsible that it is returned into store on the patient's discharge or death. A list of these articles is given in Appendix No. 14, Standing Orders for Royal Army Medical Corps.

### QUEEN ALEXANDRA'S IMPERIAL MILITARY NURSING SERVICE—*Continued.*

162E. When patients are able, she will obtain their signature on the counterfoil on Army Book 42, as an acknowledgment of having received these articles, but when patients are so ill as to be unable to look after their equipment, she will cause the ward orderly to endorse the book.

162F. When the hospital clothing and necessaries have been issued to a patient on admission, she will make an inventory of the effects which he has brought with him into hospital, and will hand these into the pack store, receiving a receipt for the same, on Army Book 42.

162G. On his discharge she will hand to the patient the receipt in her possession, in order to enable him to recover his effects from the pack store.

162H. When any case of illness or accident is brought to hospital, or in the event of any accident, emergent illness, or attempted suicide resulting in personal injury, occurring in the hospital, she will cause a medical officer to be at once informed, and, pending his arrival, will take such steps within the limits of her training as may appear to her to be necessary to meet the requirements of the case.

162I. She will be responsible that patients who have been allowed up throughout the day are in bed by 8 P.M. in winter and 9 P.M. in summer.

162J. She will see that the discharged men leave her wards in sufficient time to be present at their parade.

162K. She will visit her wards at meal times and see that the diets are properly distributed and served, and that the patients conduct themselves in an orderly manner. She will communicate any irregularity to the orderly non-commissioned officer.

162L. Sisters are earnestly requested to interest themselves in the home circumstances of men being invalided as permanently unfit, and make such representations as may be necessary to the matron for the information of the officer in charge.

163. The sister will daily receive from the steward the wines, spirits, or malt liquor ordered for the patients in her wards, and be responsible for their correct distribution, in accordance with the orders of the officers.

163A. When the daily diets and extras have been entered on the diet sheets by the officers, she will complete and sign the Diet and Extra Sheet Summary (Army Form F 734). She will then check and countersign these forms and transmit them to the steward.

164. She will take over from the steward the equipment shown on the ward inventories (which will not include bedding or patient's personal equipment), and she will be responsible for the same to the quartermaster, or to the officer in charge if there is no quartermaster.

164A. She will take over from the steward the regulated quantity of bedding for each ward.

164B. Sisters must take care that there is no waste of provisions, coals, gas, water, or other articles. Hospital forms must not be used for notes, &c. They must exercise the strictest economy compatible with the adequate supply of the patients' needs in the use of mackintosh, bandages, tow, lint, cotton wool, and all surgical dressings.

164C. Sisters are responsible for the linen allotted to their respective wards and for its good condition.

165. The sister will immediately report to the officer in charge, or to the quartermaster in a hospital in which one is doing duty, all damages or deficiencies chargeable against patients and others, as well as breakages of crockery or table glass, which, when shown to be caused by accident, are, in accordance with Regulations for Army Service Corps Duties, chargeable to the public.

166. She will have charge of books issued to patients from hospital or garrison libraries, and will prevent any improper use of them. She will at once notify any damage to them to the quartermaster, or to the officer in charge of a hospital in which no quartermaster is doing duty, in order that the amount may be assessed and recovered by means of the Personal Charge Book, as laid down in Regulations for Army Medical Services.

166A. She will submit all applications from patients for writing materials, tobacco, etc., to the officer in charge of the ward for approval, and will take such requisitions and letters to be stamped to the officer in charge of the hospital for transmission to the patient's Commanding Officer. Requisitions for these articles will be made on Army Book 38.

166B. She will see that no money, articles of diet or extras, books, tracts, pictures, or unauthorised articles of equipment are introduced into the wards without the previous sanction of the officer in charge.

167. A sister must comply with the instructions of the matron and officers. She must daily report to the matron as to the condition of her wards, or of the various departments of which she is in charge. She must be careful to mention any irregularities which may have occurred, or other matters to which her attention should be directed.

167A. She must give the matron the earliest possible information of any serious cases or operations connected with her wards, or of any other matters of importance affecting the welfare of the patients under her care.

167B. If a ward sister deems a special nurse or male attendant necessary, she must immediately report the fact to the matron. At night, the night sister must make these arrangements, mentioning full particulars in the night report.

168. The sister is held responsible for reporting to the matron if any of the staff nurses serving under her are not well, and if they appear to need medical or surgical attention.

169. The sister is personally responsible for the correct measurement of all drugs employed for hypodermic injections, sleeping draughts, and strong poisons.

She will ensure that all poisons and external applicatians are kept in their appointed place, and that the special poison cupboard is kept carefully locked, and the key removed.

She will keep the keys of such store-closets and lock-up places in the wards as may be required for the carrying out of these duties.

170. A sister is prohibited from utilising the services of nursing orderlies for any but nursing and routine ward work. She will be held responsible for carrying out the prescribed courses of nurse-training, and will, by every means in her power, afford the orderlies ample opportunity of learning their duties, and will endeavour to awaken the interest of the orderlies in all that pertains to nursing.

171. She will herself take part in the nursing of all patients seriously ill.

171A. A sister must always be present (unless, under special circumstances, her presence is not required) when an anæsthetic is administered. Should the sister be off duty, and no other sister be readily available, the staff nurse in charge of the patient must give immediate notice to the matron, that the necessary assistance may be supplied.

171B. Sisters will be detailed in rotation for duty as night sister for a period of not less than two or three months, as the matron may decide. A night sister will begin duty at 9 P.M., reporting herself at the matron's office to receive instructions; she will visit the wards at stated hours during

### QUEEN ALEXANDRA'S IMPERIAL MILITARY NURSING SERVICE—*Concluded.*

the night, and oftener if necessary; and, on coming off duty, she will submit to the matron at 9 A.M. a written report on the condition of the patients.

### Staff Nurses.

172. Staff nurses will obey the orders which they receive from the matron or sisters.

172A. In the absence of a sister from a ward, whether temporarily or permanently, the senior staff nurse present will be held responsible for the performance of the duties ordinarily performed by a sister.

172B. They must scrupulously refrain from relegating an unfair share of routine ward work to the orderlies.

172C. Staff nurses must take a full share in duties which are necessary, however unpleasant, and must set an example of cheerful alacrity in attending to the patients' wants, treating every patient with gentleness and consideration. Staff nurses must pay constant attention to the personal cleanliness of the patients.

172D. When in doubt or difficulty, staff nurses will refer to the sister in charge of their ward, and will abide by her decision.

172E. Paragraphs 161A, B, C, D, E, F, and G, apply to staff nurses as well as to sisters.

173. Staff nurses are not to go into each other's rooms after 10.30 P.M. Their bedrooms are to be neat and tidy, and all lights are to be extinguished therein by 11 P.M., unless special permission be given.

### General.

174. The period of service abroad, reckoning from the date of embarkation at home, will be from three to five years, according to climate, unless such period be incompatible with the interests of the public service.

175. On a matron, sister, or staff nurse becoming non-effective, she will hand over her copy of the "Regulations for Army Medical Services, etc.," and of the "Manual for the Royal Army Medical Corps," in her possession, to the officer in charge of the hospital, who will forward them direct to the Principal Medical Officer for disposal.

### Everybody's Opinion.

**[Correspondence on all subjects is invited, but we cannot in any way be responsible for the opinions expressed by our correspondents. No communication can be entertained if the name and address of the correspondent are not given as a guarantee of good faith, but not necessarily for publication. All correspondents should write on one side of the paper only.]**

### VILLAGE NURSES IN CUMBERLAND.

"H. C. B." writes: I have been much interested in Lady Lonsdale's answer to your note concerning the disadvantages of employing village nurses instead of fully-trained district nurses. Though a district nurse and unfortunately not fully certificated myself I cannot agree with her that the village nurse or the partially-trained nurse can possibly be as "efficient as her more fully-trained sister." The more I see of district work, especially in rural districts where doctors are not at hand and where patients trust entirely to the nurse's diagnosis of the case and leave it to her to decide whether medical advice is necessary or not, the more convinced I am that it is of the utmost importance that a high degree of training should be insisted on for the district nurse; and, as a certificated midwife myself, I marvel how anyone who has not had a good general training can undertake maternity cases without fear and with invariable success. Yet, holding these opinions, I cannot but admit that Lady Lonsdale's argument as to the utter impossibility of raising sufficient funds in small villages and rural districts for the support of Queen's or other fully-trained nurses, is an unanswerable one. In your last issue you mention an Irish town which has done away with its district nurse on account of the impossibility of raising the necessary subscriptions, and in a town not far from here, where the Queen's nurse is doing excellent work and where her services are in great demand amongst the poor, even there it is with great difficulty that she is maintained, and I feel sure that if the one or two ladies to whom her existence really is due were to leave the place or were to relax their efforts the services of this nurse too would be dispensed with for lack of funds. I observe that Lady Lonsdale speaks of the inferiority of the hospital nurse with a "smattering" of district nursing to that of the nurse who has gained her experience in the district. It is quite true that district nursing is so different in style to hospital nursing that a hospital nurse, however fully trained, would be sure to find herself rather at sea at first on the district. But the Jubilee Institute has recognised this, and insists on its nurses, however fully certificated, spending six months in a district training home before assigning them posts; therefore fully-trained hospital nurses with a thorough district training into the bargain are to be had, so that on this ground even the advantage of the village nurse over the hospital nurse cannot be proved.

"Wayfarer" writes: In reference to the system of cottage nursing in Cumberland, I fear that not only do the people suffer from such a system, but the doctors must also feel the disadvantage very much. Accidents at the works where people are employed are common, and when the hospital is a long way off these cases must be nursed at home. An operation is often required as a result of the accident, and instant action is required. I am afraid that the nurse with only "nine months' training" is little fit to cope with the emergency. It must be a help to a doctor whose patients live in outlying districts, where his visits cannot be frequent, to know that they are being attended by a nurse who not only knows what to do for the patients' comfort, but is also able to read aright the symptoms which denote that an early visit is required. The trained nurse knows how to instruct the friends and to teach them what to avoid doing—often as difficult to accomplish as the instruction in a right method. The further from hospital and more scattered the district, the greater is the need of a fully-trained nurse.

### HOME-MADE HOSPITAL PRESERVES.

"J. T." writes: Having read in a recent number of The Hospital of the "Model Jam-making Matron," I wish to say that she is not unique. I am a nurse at a cottage hospital in a small borough town, where the sisters themselves make all the jam used by the patients and staff. A fortnight ago over 100 lbs. of vegetable marrow sent from the harvest festival at the parish church, was converted into the most delicious preserve, the recipe for which has for years past been much in demand. This is not the only branch of domestic economy practised in this cottage hospital; not only is all the washing managed at home, but the gardening is chiefly done by the sisters themselves, and much expense is saved to the hospital by the vegetables which are grown in their small garden. They are planted, tended, picked, cooked and eaten by members of the establishment. I have written these few words to prove that although the practice of domestic economy is dying out amongst nurses, as in every other class of society, it is not dead yet, and we hope to live to see its revival.

### NURSES AND PENSIONS.

"A Matron" writes: I am anxious to know what can be done for nurses who cannot afford to belong to the Pension Fund. Cannot some club be started to enable nurses to pay a small sum monthly, so that when disabled by sickness or old age they may have enough coming in to support them? I am a matron and a member of the Pension Fund, but some of my nurses, who have worked for long years and yet have not saved, have nothing to look forward to. Surely this is very hard, after giving up their life to such a noble work and doing good to so many?

[Cannot the institution of which our correspondent is matron make an arrangement with the National Pension Fund which would be advantageous alike to the hospital and to the nurses? The Pension Fund is able to give so many greater advantages to nurses than any other co-operation can offer, and the benefits are so substantial, that we can suggest neither a better nor a cheaper mode of making provision for disablement or old age.—Ed. Hospital.]

### A SO-CALLED NURSING HOME AT HOLLOWAY.

"Ruth Wood" writes from 90 Hammersmith Road: Reading in the daily papers about the conviction of Mrs. Roach, I felt that the friends of the patients were as much to blame as Mrs. Roach. How can anyone undertake such patients, without being at a loss, at 16s. to 21s. per week, unless the home receives public support? Patients needing nursing, board, lodging, etc., cannot be received to the satisfaction of all concerned under two guineas without outside help.

### Appointments.

[No charge is made for announcements under this head, and we are always glad to receive, and publish, appointments. But it is essential that in all cases the school of training should be given.]

Auxiliary Nurses' Society, 10 Orchard Street, London.—Miss Annie J. Hobbs has been appointed secretary. She was trained at the West London Hospital, where she was afterwards staff nurse, and nurse in charge of the out-patient and casualty department. She has since been nurse in charge of the Establishment for Invalid Gentlewomen in Harley Street, W., night sister at the Hospital for Women, Soho, and for nearly three years senior assistant matron of the Nurses' Co-operation.

Blawarthill Hospital, Yoker, near Glasgow.—Miss Alice Gray and Miss Isabel Cribbs have been appointed charge nurses. Miss Gray was trained at the Infirmary, Chichester, and the City Hospital, Birmingham, where she was afterwards staff nurse and then ward sister. She has also been matron *pro tem.* of the Bridport Dispensary and Cottage Hospital, charge nurse at the Rural District Hospital, Greenside, Sheffield, and has had experience in private nursing. Miss Cribbs was trained at the Belvidere Hospital, Glasgow, and then held the post of charge nurse at the Blawarthill Hospital, Yoker. She has since been charge nurse of the diphtheria block at Hastings Sanatorium, and private nurse on the staff of the Sunderland Nursing Institute.

Bradford Eye and Ear Hospital.—Miss Jeannie Brooke has been appointed sister. She was trained at the Sheffield Royal Infirmary and has since been nurse at the Sheffield Borough Sanatorium for Infectious Diseases, charge nurse at the Parkhill Hospital, Liverpool, and charge nurse at the Royal Infirmary, Wigan.

Cheltenham Union Infirmary.—Miss Annie Phillips has been appointed charge nurse. She was trained at the Stapleton Infirmary, Bristol, having previously been sixteen months at Atcham Union Infirmary. She holds the L.O.S. certificate.

Chepstow Workhouse.—Miss Ada Wordthorpe has been appointed head nurse. She was trained at Cork Street Fever Hospital, Dublin, Drumcondra General Hospital, Dublin, and, for maternity, at the Abbey Hospital, Paisley. She has since been head nurse at Gainsborough Union Infirmary.

Colne Jubilee Cottage Hospital.—Miss Billinge has been appointed staff nurse. She was trained at the Crumpsall Infirmary and at the Manchester Maternity Hospital. She holds the L.O.S. certificate.

Indian Army Nursing Service.—Miss Aimée M. Cockcraft, Miss Isabel K. Grant, Miss Anna J. Marshall, Miss M. S. Pocock, and Miss Mary C. Quinn have been appointed sisters. Miss Cockcraft was trained at Addenbrooke's Hospital, Cambridge, and has since been staff nurse at the Royal Hospital for Children and Women, Waterloo Road, London, S E. She has served on plague duty in India, and since April, 1901, on the Army Nursing Service Reserve at the Royal Victoria Hospital, Netley. Miss Grant was trained at the London Hospital, where she has since been sister. Miss Marshall was trained at St. George's Hospital London, and has served on plague duty in India since 1899. Miss Pocock was trained at the London Hospital, where she has since been sister. Miss Quinn was trained at St. Vincent's Hospital, Dublin. She has since done private nursing in connection with the Leicester Institute, and from February, 1900, has served on the Army Nursing Service Reserve at the Royal Victoria Hospital, Netley.

Jessop Hospital for Women, Sheffield.—Miss F. C. Cartwright has been appointed sister. She was trained at Guy's Hospital, and in midwifery at Plaistow Maternity Hospital.

Mount Vernon Consumption Hospital, Hampstead.—Miss Zoë Longstaff has been appointed matron. She was trained at the London Temperance Hospital, and was a special probationer at the Women's Hospital, Soho Square, London. She has since been holiday charge nurse at Friedenheim Home for the Dying, Swiss Cottage, sister of the Male and Female Wards of the National Hospital for the Paralysed and Epileptic, Queen Square, London, and for the last three years senior sister at the Mount Vernon Consumption Hospital.

Newport (Monmouthshire) County Hospital.—Miss May Godson has been appointed sister. She was trained at the Queen's Hospital, Birmingham, and has since been sister at the Infirmary, Shirley, Southampton.

St. Mary's Hospital for Sick Children, Plaistow.—Miss M. R. S. Asquith has been appointed matron. She was trained at St. Bartholomew's Hospital, London, and has since been sister at the East London Hospital for Children, Shadwell, E., and matron of the Children's Branch of the Poplar and Stepney Sick Asylum.

Sherburn Hospital, near Durham.—Miss Edith Anne Weale has been appointed matron. She was trained at the Royal Southern Hospital. Liverpool, where she was afterwards sister. She has since been matron, for more than seven years, of Skinningrove Hospital.

Swansea General and Eye Hospital.—Miss Mary Coverdale has been appointed sister. She was trained at the Norfolk and Norwich Hospital, and has since been sister in the same institution. She has also done private nursing.

West Norfolk and Lynn Hospital.—Miss Marie Ethel Wood has been appointed charge nurse. She was trained at the County Hospital, Newport, Mon., where she was afterwards night superintendent. She has since, for the last eighteen months, been engaged in private nursing.

### Echoes from the Outside World.

### The King in London.

The King left North Berwick on Saturday after a couple of days' stay, having carried out a very full programme on the Friday. He was out early, accompanied by his host, Prince Edward of Saxe-Weimar, and proceeded to the golf links, where he had a few minutes' conversation with Mr. Ben Sayers, the professional golfist, whom he commanded to supply for him a complete set of clubs. Upon his return to North Berwick he planted a tree, a purple leaved sycamore, before the Council Chamber, in commemoration of his visit to the town. He accepted as a gift the silver bladed spade with which he performed the ceremony. His Majesty then returned to lunch at the Knoll, and afterwards planted another tree, this time a fir, in the grounds of his host's residence. In the afternoon he went for a long motor ride of 30 miles with Mr. Balfour in the Prime Minister's powerful motor, and repaired to Lord Haddington's house, Tyningham, to tea. Here he planted a third tree, an oak, and accepted the gift of a second spade. In the evening he returned to the Knoll in time to dine and witness the illuminations of the town. Upon His Majesty's arrival at King's Cross on Saturday—ten minutes' stay having been made at Newcastle to allow of the King signing certain important documents—he was accorded an enthusiastic welcome. On Monday the King entertained Lord Kitchener and Lord Roberts to lunch at Buckingham Palace, and several American officers were also invited. Lord Kitchener leaves this week to take up his command of the military forces in India.

### Movements of the Queen.

Queen Alexandra has derived much enjoyment during the past week from various musical entertainments in Copenhagen. She sent for Mr. Anton Hegner, the American 'celloist, heard him play, presented him with three gems—a ruby, a diamond, and a sapphire—representing the British colours. At a private concert a little later Queen Alexandra and the Dowager-Empress of Russia played the piano, the Hereditary Grand Duke of Russia performed on the flute, other members of the Danish Royal Family singing or playing their favourite instruments. The following evening a visit was paid to the Theatre Royal to hear "Cavalleria Rusticana." On Monday the members of the Royal Family visited the Russian cruiser *Boyarin*, which was built at Copenhagen. The whole party then repaired to the Russian Imperial yacht and lunched there. The Queen has asked for and obtained a list of those Danes who took part in the South African war on the British side. Her Majesty is expected to return to London on or about the 22nd inst.

### The Gladstone Statue at Glasgow.

At Glasgow, on Saturday, Lord Rosebery unveiled a statue, erected at a cost of some £4,000 by the citizens of Glasgow, to the memory of Mr. Gladstone. The statue is in bronze, and is by Mr. Hamo Thorneycroft, R.A. It represents the distinguished statesman in his robes as Lord Rector of Glasgow University. There are two panels on the pedestal—that on the south side representing Mr. Gladstone addressing the House of Commons, while that on the north depicts him felling a tree in the grounds of Hawarden. At the close of an address of remarkable eloquence, Lord Rosebery said that history had not yet allotted Mr. Gladstone his definite place, but no one could now deny that he bequeathed a pure standard of life, a record of lofty ambition for the public good as he understood it, a monument of life-long labour. "Such lives," continued Lord Rosebery, "speak for themselves. They need no statues. They face the future with the confidence of high purpose and endeavour. The statues are not for them—but for us—to bid us be conscious of our trust, mindful of our duty, scornful of opposition to principle and faith. They summon us to account for time and opportunity; they embody an inspiring tradition; they are milestones in the life of a nation. The effigy of Pompey was bathed in the blood of his great rival; let this statue have the nobler destiny of constantly calling to life worthy rivals of Gladstone's fame and character."

### The New Dean of Westminster.

The new Dean of Westminster has been appointed. Canon Armitage Robinson succeeds Dr. Bradley. The Premier has also nominated to the canonry, vacated by the promotion of Dr. Robinson, the Rev. Henry Charles Beeching, Professor of Pastoral and Liturgical Theology at King's College, and Chaplain of Lincoln's Inn. Both the new dignitaries are eminent scholars as well as excellent preachers, and neither of them has identified himself with any particular party. Dr. Robinson was domestic chaplain to Bishop Lightfoot for a year. He has been examining chaplain to the Bishop of Bath and Wells, and Norrisian Professor of Divinity at Cambridge. He is also the author of many learned books. Professor Beeching will be the youngest member of the Chapter except Canon Henson, and has had parochial experience at Liverpool and in Berkshire. But his title to fame rests largely upon his volume of delightful poems and his brilliant sermons. The author of "Love in Idleness" can scarcely fail to attract attention as Canon of Westminster.

### Air-ship Disaster in Paris.

Experiments in the art of aerial navigation continue to be marked by sad loss of life. It is only five months since M. Severo and his assistant were killed in Paris in their balloon Pax; now a Hungarian nobleman and his French companion have met with their deaths in the same city making an initial trip in the new air-ship they had invented. In the case of the Brazilian inventor the machine exploded owing to the motor being near to the petrol tank; in the accident on Monday the platform upon which Baron de Bradsky and his electrical engineer were standing broke away from the balloon and the occupants were dashed to the ground. Apparently the wires which bound the two portions of the machine together were not strong enough to bear the strain. Baron de Bradsky was only 36 years of age, very wealthy, and he had left the Diplomatic Service to devote himself to ballooning, over which he displayed much enthusiasm. He leaves a wife (who was present at the ascent) and three children, the youngest only four years of age. M. Morin was also married and leaves a daughter of 13.

### Women Workers.

The Conference of the National Union of Women Workers will be opened at St. Cuthbert's Hall, Edinburgh, on the 28th of this month, by Lady Battersea, who will deliver the presidential address. The rest of the morning will be devoted to discussion concerning University education for women and its effects on social and intellectual life. "The Present Position of Women's Suffrage" and "Public-house Trusts" will be amongst the subjects of the papers read. On the second day Lady Frederick Cavendish will preside over a meeting at which the question of "The Permanent Care of the Feeble-minded" will be debated, also "Rescue Work in Scotland and amongst Children"; but perhaps just now the greatest interest will attach to the paper on "The Outlook for Women at Home and in the Colonies," which is certain to call forth valuable arguments, both in favour of female emigration and otherwise.

### A Book and its Story.

### MISS CAREY'S NEW STORY.*

Nothing can be more charming, in its way, than this latest story from Miss Carey's untiring pen. Fresh in its tone and attractive to a degree, in the delineation of scenes and people who are quite natural and therefore, of necessity interesting, it will be read with satisfaction by those who appreciate these wholesome qualities. An absolutely well-drawn character is Miss Durnford known affectionately to her friends by the informal title of "Miss Jem." It is one that suits the kindly, uncultured, wealthy woman, whose good heart made up for the deficiencies arising from the circumstances of her birth and her lack of education; and who lived in lively awe of a household devoted to its mistress, in spite of a certain tyranny which asserted itself in situations in which its members considered that length of service entitled them to decide what was best for "Miss Jem." Being a woman of will, if she deferred on lesser occasions to their suggestions, it was only with the intention of following up on more important ones the leading of her own mind. She had for some time been disturbed by the question of taking a companion. "You want a nice, cheerful young lady to keep you company," a friend had said one day when she was confessing to a feeling of loneliness. She acted on the suggestion, and lost no time in going up to town from the charming old place in the country, bought for her by her late father, after his retirement from business. She was not long in meeting with someone who came up to the easy standard fixed by her friend, and who, in addition, possessed an unfailing fund of common sense to balance an unselfish and deep-feeling nature. Eunice Cleveland, on whom "Miss Jem's" choice fell, was the sister of Dr. Cleveland, a medical man, with a growing family, and an income limited by its association in practice with a partner who was old in years and methods. She had lived with her brother and sister-in-law since her parents' death, and in return for her home had made herself useful to them in any way possible. But she realised, as the family grew larger, that even her presence was a tax on her brother's income, which she could no longer impose. So she decided, much against his wish, to do something for herself. Having replied to a letter from Miss Durnford, offering her the post of companion and appointing a meeting at the Métropole, she went there at the time arranged, to find "a lady who was not so bad-looking after all, as she plumped herself down in the opposite chair and wrestled with her tight gloves. " . . . When Eunice knew her better she was often amused by her childish and transparent vanity and her love of finery." On this occasion Miss Durnford's toilette, an otherwise harmonious one, was spoilt by an exceedingly smart hat trimmed with glaringly contrasting colours. "I am a regular peacock in my tastes," she would say, "I am so fond of bright colours. Susan, who is a dissenter and only likes drabs and browns, often warns me that dress, not money, is the root of all evil. She will give me a rare scolding when she opens the parcels, and if there is anyone I am afraid of it is Susan." Miss Durnford being attracted by Eunice's appearance announced with characteristic brevity: "Why the moment I set eyes on you I said to myself, Miss Cleveland will do, she looks sensible and good-humoured, and if I am not wrong she will suit me down to the ground as sure as my name is Jemima Anne Durnford." This sensible and good-humoured young woman found her work cut out for her in defending "Miss Jem" from the promptings of an over-generous heart, which caused her to act impulsively and in a way not always to her own interest, or that of the person whom she wished to befriend. "She is dreadfully incautious. She is nine and thirty and has no more knowledge of the world than I have; not so much I fancy." Eunice found that Miss Durnford's place was in every way delightful. To her limited experience it was "magnificent and altogether palatial." The hall was large, and full of handsome oak furniture, and the drawing-room, library, and morning room were all well proportioned and cheerful. The drawing-room was charming with its air of old-fashioned cosiness. When she showed her delight by exclaiming "What a beautiful room!" Miss Durnford replied, "There now, that's what people always say . . . but I never could abide silver tables and Japanese fans, and all that Liberty nonsense." She disclaimed any individual taste in the arrangement of the house; she gave praise where it was due, to the late owner.

In descriptions of scenes of country life and society, Miss Carey is particularly at home. She writes of them with the same lively interest, but with more imagination, than Miss Austin, to whom she is a worthy successor. Mrs. Pater, an incorrigible mimic, is a good delineation. She could not refrain from taking off her friends, even to their faces. "She was so diminutive that for a moment Eunice thought she was a child, and she was dressed like a little robin in furry red." She was in hiding in Miss Durnford's drawing-room one evening as it was growing dusk. "Lor, now Mrs. Pater," exclaimed Miss Durnford, "it is like you to be masquerading in the dark, when most folks are thinking of their dinner." Miss Jem's summary of Mrs. Pater's peculiarities was given in reply to a question of Eunice's "as to whether a dinner party to which they were asked would be a large one? I could not quite understand Mrs. Pater." "I daresay not," returned Miss Jem, "you never can be up to her tricks. She may have asked the whole neighbourhood with a band and a pastrycook supper, or she may only have a dozen of the dowdiest old ladies in Desborough—her tabbies as she calls them."

Eunice found her life particularly happy. The Hiltons were their nearest neighbours, a brother and his sister living at Friar's Farm. Eunice's first visit to them was one of unending interest to her. "There are some days in everyone's life—of which this was one—which deserve to be laid up in lavender and placed in the snuggest corner of one's mental storeroom, or strewn with sweet, dry rose leaves—days which even to the memory distil a faint, far-off fragrance." The old sundial at Friars' Farm expressed this sentiment. "*Horas non numero, nisi Serenas*"—"I only note the sunny hours," or, as Lilian Hilton preferred to translate it, "I never count the hours that do not bring me peace." Friar's Farm had been a monastery. "The old refectory was a pleasant place that evening with its blazing fire and soft lamp-light shining on the gleaming silver and beautiful old-fashioned china of Lilian's tea-table. Nigger, the dog, was stretched on a tiger skin in front of the fire, some softly-cushioned chairs and the oak settle looked deliciously inviting, while the tall screen shut in the circle of brightness from the haunting shadows that appeared to lurk at the other end of the hall, the great rafters and dark panelling seeming to swallow up the light." Comedy with a mere suggestion of tragedy by the introduction of an artist with a history, and his little motherless boy who excite more than a passing interest in Aunt Jane's warm heart is the dominant note of the "Highway of Fate." Eunice has her friends and admirers. Douglas Hilton was engaged when they first met, and felt for each other a mutual attraction. After a short married life he was left a widower. Years passed on, his marriage had not been a happy one, and when he meets Eunice again he decides that it shall not be as it once was with them. "Then he and Eunice had passed each other sadly on the highway of fate and with silent hopelessness had each taken their separate paths, but now the changes and chances of life had brought them together again. With the spring a sweet young mistress would come to Friar's Farm to be his wife and helpmeet." The interest of the story is sustained to the end.

* "The Highway of Fate." By Rosa N. Carey. (I Vol. Price 6s. Publishers: Macmillans.)

### For Reading to the Sick.

ST. LUKE'S DAY.

Two clouds before the summer gale
  In equal race fleet o'er the sky;
Two flowers, when wintry blasts assail,
  Together pine, together die.

Two converts, watching by his side,
  Alike his love and greetings share;
Luke the beloved, the sick soul's guide,
  And Demas named in faltering prayer.

Pass a few years—look in once more—
  The saint is in his bonds again;
Save that his hopes more boldly soar,
  He and his lot unchanged remain.

But only Luke is with him now:—
  Alas! that even the martyr's cell,
Heaven's very gate, should scope allow
  For the false world's seducing spell.

*Keble.*

---

With the waning year comes the day of St. Luke, the beloved physician. At the parting of the ways it stands while summer fragrance still lingers in the air and Nature puts on her autumn glory of gold and red. The harvest is gathered in, the days are shortening, and everything speaks of the approach of the resting time for which mother earth is making ready. Then, just when we are preparing to face the grey November days, we are gladdened by a brief summer interlude, a little angel's visit, when the balmy air, and fleecy clouds, and late roses still blowing, make us for the moment, dream that it is June again. No, it is only the "Summer of St. Luke," true son of consolation, and we welcome his festival at a time, when with playful dalliance, Nature re-assumes a summer mood, and we too rejoice with her and keep in mind St. Luke, beloved of Our Lord, and faithful.—*Anon.*

---

It is not certain that St. Luke died a martyr; but we cannot doubt that he lived a saint.

Setting aside a question easily raised and not easily answered, whether the "Luke" or "Lucas" named three times by St. Paul is or is not this Evangelist, and assuming such identity, we notice how very tenderly he is mentioned as "Luke, the beloved physician:" and again with a brevity more expressive than a multitude of words, "Only Luke is with me."

But in St. Luke's Gospel, and in his Book of Acts, his own name occurs not so much as once. In the Gospel it seems impossible to trace him, except perhaps by help of tradition: in the Acts we infer his presence on certain occasions only from his use of the word "we" and its derivatives.

Thus St. Luke illustrates for our edification one of King Solomon's noble proverbs: "Let another man praise thee, and not thine own mouth; a stranger, and not thine own lips."—*C. Rossetti.*

---

Ah! dearest Mother, since too oft
  The world yet wins some Demas frail
Even from thine arms, so kind and soft,
  May thy tried comforts never fail!

When faithless ones forsake thy wing,
  Be it vouchsafed the still to see
Thy true, fond nurslings closer cling,
  Cling closer to their Lord and thee.

### Notes and Queries.

**The Editor is always willing to answer in this column, without any fee, all reasonable questions, as soon as possible.**

**But the following rules must be carefully observed:—**

**1. Every communication must be accompanied by the name and address of the writer.**

**2. The question must always bear upon nursing, directly or indirectly.**

**If an answer is required by letter a fee of half-a-crown must be enclosed with the note containing the inquiry, and we cannot undertake to forward letters addressed to correspondents making inquiries. It is therefore requested that our readers will not enclose either a stamp or a stamped envelope.**

*Abroad.*

(12) I am anxious to go to Smyrna as a private nurse. Will you kindly let me know if there is an opening for such, and to whom I should apply?—*K. S.*

Perhaps the Bible Lands Missions' Aid Society, 7 Adam Street, Strand, W.C., could give you information.

*Rules.*

(13) I am desirous of knowing the rules of charitable nursing associations, and shall feel obliged for any information.—*W. B.*

Write to the General Superintendent, Queen Victoria's Jubilee Institute for Nurses, St. Katherine's Precincts, Regent's Park, N.W. A part of the very valuable work done by the Queen's nurses is the aid given in the formation and management of institutions for nursing the poor.

*Blind.*

(14) Can you tell me if there is a benevolent society for the blind, or any charity which allows a weekly sum to a blind person?—*Bertha.*

You will find in "Burdett's Hospitals and Charities" a long list of institutions for aiding the blind. It is quite impossible to mention the name of one just suited to any particular case, especially when no particulars are given.

*Skeleton and Skull.*

(15) Can you tell me where I can purchase a disarticulated skeleton and skull for the purposes of studying?—*K. G. G.* and *Nurse.*

Your local chemist or surgical instrument maker could obtain them for you. Write to the Midwives' Institute, 12 Buckingham Street, Strand, as some of their members may have the bones for sale.

*Hospital Training.*

(16) Will you kindly tell me what is the shortest time in which I could gain a certificate in general nursing from an institution where no menial work would be required, and what the terms would be?—*K. L.*

One year, and the terms would be about one guinea per week. See "The Nursing Profession: How and Where to Train."

*Up-Country Nursing Association.*

(17) Will you kindly tell me to whom I should apply concerning the vacancies of the Up-Country Nursing Association for Europeans in India?—*Sister* and *Nurse A. F.*

Mrs. Sheppard, 10 Chester Place, Regent's Park, N.W.

*Sanatorium.*

(18) Will you kindly tell me of a sanatorium at Bournemouth where a poor woman, believed to be curable, may be treated for phthisis either free or a very little cost?—*Phthisis.*

The National Sanatorium for Consumption at Bournemouth has a few free beds. Write for particulars to the Secretary.

**Useful Handbooks for Nurses.**

"Nurses' Dictionary of Medical Terms." Cloth, 2s.; leather, 2s. 6d.; post free 2s. 8d.
"On Preparation for Operation in Private Houses." 6d.
"Hospital Sisters and their Duties." 2s. 6d.
"Medical Gymnastics, including the Schott (Nauheim) Movements." 2s. 6d.
"The Human Body." 5s.
"Practical Handbook of Midwifery." 6s.
"A Handbook for Nurses." (Illustrated.) 5s.
"Tendencies to Consumption: How to Counteract Them." 2s. 6d.
"Syllabus of Lectures to Nurses." 1s.

The above works are published by the Scientific Press, Ltd., and may be obtained through any bookseller or direct from the publisher, 28 and 29 Southampton Street, Strand, London, W.C.

## Figures and Tables

**Figure f1:**